# Congenital myopathies: clinical phenotypes and new diagnostic tools

**DOI:** 10.1186/s13052-017-0419-z

**Published:** 2017-11-15

**Authors:** Denise Cassandrini, Rosanna Trovato, Anna Rubegni, Sara Lenzi, Chiara Fiorillo, Jacopo Baldacci, Carlo Minetti, Guja Astrea, Claudio Bruno, Filippo M. Santorelli, the Italian Network on Congenital Myopathies, Angela Berardinelli, Enrico S. Bertini, Giacomo Comi, Adele D’Amico, Maria Alice Donati, Maria Teresa Dotti, Fabiana Fattori, Marina Grandis, Lorenzo Maggi, Francesca Magri, Maria A. Maioli, Alessandro Malandrini, Francesco Mari, Roberto Massa, Eugenio Mercuri, Luciano Merlini, Maurizio Moggio, Marina Mora, Lucia O. Morandi, Olimpia Musumeci, Vincenzo Nigro, Marika Pane, Elena Pegoraro, Elena M. Pennisi, Lorenzo Peverelli, Giulia Ricci, Carmelo Rodolico, Lucia Ruggiero, Michele Sacchini, Lucio Santoro, Marco Savarese, Gabriele Siciliano, Alessandro Simonati, Paola Tonin, Antonio Toscano

**Affiliations:** 1 Molecular Medicine, IRCCS Fondazione Stella Maris, Pisa, Italy; 2 Neurology, IRCCS Fondazione Stella Maris, Pisa, Italy; 3 Unit of Pediatric Neurology and Muscular Disorders, Istituto G. Gaslini, Genoa, Italy; 4 Department of Neuroscience, Center of Myology and Neurodegenerative Disorders, Istituto G. Gaslini, Genoa, Italy; 5 Department of Neuroscience, Rehabilitation, Ophthalmology, Genetics, Maternal and Child Health, University of Genova, Genoa, Italy

**Keywords:** Congenital myopathy, Next generation sequencing, Muscle MRI, Muscle biopsy

## Abstract

Congenital myopathies are a group of genetic muscle disorders characterized clinically by hypotonia and weakness, usually from birth, and a static or slowly progressive clinical course. Historically, congenital myopathies have been classified on the basis of major morphological features seen on muscle biopsy. However, different genes have now been identified as associated with the various phenotypic and histological expressions of these disorders, and in recent years, because of their unexpectedly wide genetic and clinical heterogeneity, next-generation sequencing has increasingly been used for their diagnosis. We reviewed clinical and genetic forms of congenital myopathy and defined possible strategies to improve cost-effectiveness in histological and imaging diagnosis.

## Background

The term congenital myopathy refers to a group of clinically, genetically and histologically heterogeneous diseases that mainly affect muscle tissue. The presence of particular histopathological alterations on muscle biopsy distinguishes these conditions from other neuromuscular disorders. Congenital myopathy is caused by genetically determined defects in structural proteins of muscle and classified on the basis of muscle biopsy findings [1]. The onset generally occurs in the neonatal period. Although the precise epidemiology of congenital myopathy is not known, it has an estimated incidence of around 1:25,000, and has been reported to account for 14% of all cases of neonatal hypotonia [2]. Although the classification of congenital myopathy is under constant review as more genes are identified and associated with its various phenotypic and histological expressions, for the moment it continues to be based mainly on the features seen on muscle biopsy. Accordingly, congenital myopathy can be divided into the following five forms:

1. nemaline myopathy (subtypes: rod, core-rod, cap and zebra body myopathy);
2. core myopathy (subtypes: central core and multiminicore myopathy);
3. centronuclear myopathy (subtypes: myotubular myopathy and autosomal centronuclear myopathy);
4. congenital fiber-type disproportion myopathy;
5. myosin storage myopathy

This paper describes the different congenital myopathy disease types, focusing, in particular, on their diagnosis through muscle biopsy, their muscle MRI features, and the use of genetic testing based on cutting-edge gene analysis technologies (next-generation sequencing, NGS). Although adult-onset sporadic nemaline myopathy, spheroid body myopathy, sarcotubular myopathy and reducing body myopathy were all initially regarded as forms of congenital myopathy, they were recently excluded from the official classification [1] on the basis of expert opinion, which deemed that they may be more appropriately grouped with other neuromuscular disorders. For example, spheroid body myopathy caused by mutations in *TRIM32* and sarcotubular myopathy caused by mutations in *MYOT* may more correctly be grouped with the limb-girdle muscular dystrophies.

## Clinical characteristics

Clinical phenotype, in isolation, remains an inadequate basis for distinguishing between the different types of congenital myopathy, as it is often poorly specific, usually consisting of hypotonia and weakness (present at birth or appearing in infancy) and a static or slowly progressive clinical course. The clinical spectrum is nevertheless recognized to range from severe neonatal forms with congenital arthrogryposis to mild childhood-onset forms with non-progressive hyposthenia and low muscle tone [1, 3].

In neonatal and very early-onset cases, the symptoms are generally more pronounced, and may consist of reduced fetal movements and subsequent development of arthrogryposis and clubfoot. Severe muscle hypotonia is often present at birth and in the early months of life (floppy baby sign), together with a frog-like posture, difficulty sucking, and respiratory insufficiency. Other typical symptoms in the first year of life are congenital hip dysplasia and hypomimia (myopathic facies). As the child grows, the muscle hypotonia remains stable, while motor milestones are delayed; bone and joint deformities may appear, such as joint contractures, lordosis, scoliosis or a rigid spine. A significant loss of muscle mass with a low body weight and diffuse muscle atrophy are other common features [4].

Patients with congenital myopathy typically show dysmorphic facial features secondary to muscle weakness, associated with an arched palate, micrognathia and *pectus carinatum* or *excavatum*, the latter resulting from chest muscle hypotonia. Abnormal extrinsic eye movements with eyelid ptosis and strabismus may develop later on. Ophthalmoparesis is a frequent clinical feature in some forms.

### Nemaline myopathy

Nemaline myopathy is characterized by the presence of small rod-like inclusions in muscle fibers. Made up mainly of alpha-actinin, actin and other Z-band filaments, these inclusions are clearly visualized by Gomori trichrome staining [5].

Nemaline myopathy has an incidence of 1:50,000 live births, although it may be more common in certain populations (e.g. in Ashkenazi Jews, or in the Amish community).

The clinical spectrum of nemaline myopathy is quite broad, ranging from mild to severe phenotypes [6].

Children affected by severe, neonatal-onset forms, which account for around 16% of all cases, are hyposthenic, hypotonic from birth, and have difficulty sucking and swallowing. They may present arthrogryposis and dilated cardiomyopathy. Respiratory failure or aspiration pneumonia will often lead to death in the first weeks or first months of life.

The most common form, however, is less severe, being characterized by weakness of the limbs, trunk and facial muscles, and by a stationary or slowly progressive disease course. Adaptation to extrauterine life is adequate, but the acquisition of motor milestones is delayed. Muscle hypotonia is always present during the first year of life. Weakness of the head muscles is associated with distinctive facial features (an elongated face, high-arched palate and tented upper lip) and with dysarthria and dysphagia. Although children affected by this form of myopathy develop muscle contractures and high-arched feet, most are able to walk. Respiratory involvement is the main prognostic factor [7].

One specific subtype of nemaline myopathy is cap myopathy, a very rare congenital type. Only 20 patients have been described since its first description in 1981 [8]: 13 with a sporadic and seven with a familial form [9–13]. Cap myopathy has neonatal or childhood onset and its progression is slow. Affected individuals present weakness and hypotrophy of axial and proximal muscle groups, a long face with high-arched palate, scoliosis and breathing difficulties.

Zebra body myopathy is a benign congenital myopathy, characterized by congenital hypotonia and weakness. Its prevalence is unknown; fewer than ten patients have been described so far. Muscle biopsy shows zebra bodies and other myopathic changes.

### Core myopathy

The term core myopathy refers to forms of congenital myopathy characterized histologically by the presence, in muscle cells, of rounded structures called “cores” in which oxidative activity ranges from markedly reduced to absent, unlike the surrounding area where it is normal. On the basis of differences between these structures, as seen on biopsy, two separate entities have been designated [14, 15]: central core myopathy is defined by the presence of individual cores that are usually centrally located, while multiminicore myopathy is morphologically characterized by the diffuse presence of numerous small cores (multiminicores). The prevalence of these forms is unknown.

The two core myopathy subtypes are the most common forms of congenital myopathy and clinically they are highly heterogeneous. Central core myopathy is inherited as an autosomal dominant or autosomal recessive trait with variable penetrance [16]: its clinical severity can vary between members of the same family and also with age. Autosomal recessive forms are clinically more severe and there exist descriptions of patients with fetal akinesia and congenital arthrogryposis [17]. With the exception of these latter, neonatal-onset cases, core myopathy has a fairly benign disease course. The symptoms include: delayed walking, occasional involvement of the muscles of facial expression, reduced eye movements, eyelid ptosis and hypotonia and weakness of the proximal and axial muscles. Respiratory function is well preserved. There have been rare reports of infants presenting with a more serious clinical picture, including early respiratory failure [18]. Finally, some patients may show only susceptibility to malignant hyperthermia and raised serum creatine kinase (CK) levels.

The clinical characteristics of multiminicore myopathy, too, can vary greatly, and they are, in part, genetically determined [19]. Onset is usually in childhood or the late teens but adult forms also exist. Four phenotypic forms have been described, albeit with considerable overlap of symptoms between them [20]. Patients with the most common phenotype (classic form) present severe axial muscle weakness with scoliosis or stiffness of the spine, torticollis and respiratory involvement. However, the degree of muscle weakness is not always correlated with the severity of the respiratory insufficiency. The progression of the symptoms is slow and these patients may present a clinical overlap with rigid spine congenital muscular dystrophy. In a second group of patients, complete or partial external ophthalmoplegia is present in addition to the muscle involvement [21]. A third, small, group of patients presents proximal weakness, typically of the pelvis or shoulder girdle, associated with arthrogryposis. Finally, some cases with a severe neonatal presentation have been described in the literature [22]. In all these forms of multiminicore myopathy, the clinical manifestations usually have early onset and CK levels are normal or only slightly raised. Some patients may experience muscle pain on exertion.

### Centronuclear myopathy

The key characterizing feature of the forms collectively referred to as centronuclear myopathy is the abnormal position of the nuclei within muscle cells, i.e. they are often exactly in the center of the cells. Furthermore, they are larger than normal and have a vesicular appearance, making them reminiscent of myotubes; hence the origin of the term myotubular myopathy, which is the name of one of the subtypes [23].

The term myotubular myopathy refers only to the X-linked form of the condition (XLMTM), while the term centronuclear myopathy is normally used to indicate the autosomal form, inherited as a dominant or, more rarely, recessive trait [24]. According to the findings of a recent review of Italian patients [25], around 30% of centronuclear myopathy cases remain genetically undiagnosed, which suggests that there are still new disease-causing genes to be identified.

#### Myotubular myopathy

The X-linked form of centronuclear myopathy has an estimated incidence of around 2:100,000 male births [26, 27]. The first symptoms may even appear in the womb, consisting of polyhydramnios and reduced fetal movements. Affected subjects, usually boys, present at birth with hypotonia and respiratory insufficiency. They can also have multiple arthrogryposis with spinal and rib cage deformities. Dysphagia is a major problem and a nasogastric tube is often needed for feeding. Reduced eye movements and eyelid ptosis are common features. Patients are often macrosomic and may have other malformations such as pyloric stenosis, inguinal hernias and cryptorchidism. They generally die during the first year of life due to respiratory insufficiency or aspiration pneumonia. Only a small percentage of affected subjects reach adolescence or adulthood, and these cases require assisted mechanical ventilation and gastrostomy for feeding [28].

Exploration of the medical history may disclose a history of repeated miscarriages of male fetuses on the mother’s side of the family. There are no reports of cardiomyopathy, cardiac conduction defects or arrhythmias [29].

Female carriers are generally asymptomatic or may occasionally show, in childhood, mildly progressive limb-girdle weakness and hypomimia [30]. The presence of muscle symptoms in female carriers seems to be linked to an X chromosome inactivation defect [31, 32].

#### Autosomal centronuclear myopathy

Compared with the X-liked form, autosomal centronuclear myopathy shows a greater genetic heterogeneity and a later onset, i.e. in childhood or early adulthood. Cases with onset in childhood present with hypotonia, distal/proximal muscle weakness, rib cage deformities (sometimes associated with respiratory insufficiency), ptosis, ophthalmoparesis and weakness of the muscles of facial expression with dysmorphic facial features; the motor milestones are reached, but delayed.

The adult form presents with moderate proximal muscle weakness, which never becomes severe; the disease progresses slowly and affected individuals seem to have a normal life expectancy, although loss of ambulation is possible after 50 years of age.

Serum CK is normal or only mildly elevated, while electromyography (EMG) shows non-specific myopathic abnormalities.

### Congenital fiber-type disproportion myopathy (CFTD)

In CFTD, the main histological abnormality (from which the condition gets its name) is a disproportionate difference in fiber caliber between the type 1 (slow) and type 2 (fast) fibers, with the type 1 fibers found to be significantly and consistently smaller than the type 2 ones. The prevalence of CFTD is unknown. Most affected children present with hypotonia and mild-to-severe generalized muscle weakness at birth or within the first year of life. Motor milestones are delayed, head control is poor, and the muscle weakness is particularly evident facially and at the pelvis and shoulder girdle. Deep tendon reflexes are reduced or absent. In more than 90% of affected individuals, the muscle weakness is static but the overall disease course is slowly progressive [33].

At least 30% of those affected by CFTD present significant respiratory muscle hypotonia, which leads to defective functioning of the chest bellows. The disease can occur at any age; some children are affected by CFTD from infancy, and these cases show severe respiratory involvement, but this does not necessarily mean a poor prognosis. Some patients, on the other hand, develop mild respiratory failure which causes nocturnal hypoxia and hypercapnia resulting in symptoms such as morning headaches, daytime fatigue, decreased appetite, weight loss, difficulty sleeping and frequent lung infections.

Dysphagia is present in 30% of children with CFTD. Chewing and swallowing difficulties can cause ingestion of food, liquid, saliva or other material into the lungs and lead to aspiration pneumonia. As in other forms of congenital myopathy, dental crowding and a high arched palate are typical features. Infants with severe bulbar weakness may have feeding problems that, if they persist beyond the first months of life, require intervention (enteral feeding and/or gastrostomy). The mildest feeding problems often subside over time.

Contractures may be present at birth or occur in older children as a result of decreased mobility due to muscle hypotonia. Contractures of the ankles, fingers, hips, elbows and knees occur in about 25% of affected children. Congenital hip dislocation and clubfoot may also be present. Hypotonia and hypotrophy of the paraspinal muscles causes rotation of the vertebral bodies resulting in scoliosis, kyphoscoliosis and lordosis in 25% of cases, or more. Contractures and abnormalities of the spine are not necessarily associated with greater disease severity.

### Myosin storage myopathy

This form, first reported in 1971 [34] and subsequently termed myosin storage myopathy [35], is a rare condition associated with mutations in a myosin heavy chain beta (MHC-β) isoform that is expressed primarily in the heart, but also in type 1 skeletal muscle fibers. To date, there exist descriptions of clinical phenotypes associated with hypertrophic or dilated cardiomyopathy and also with a wide range of skeletal myopathies including distal myopathy [36], myosin storage myopathy itself [35], CFTD [37] and a form of multiminicore myopathy [38]. Cardiac involvement usually occurs in isolation, but can also appear in association with other forms of congenital myopathy [39]. The clinical manifestations are highly variable even within single families. Onset usually occurs in childhood, with delayed motor milestones. Calf pseudo-hypertrophy and foot drop are common features. Lack of strength in the wrists and hands is a major feature of Laing distal myopathy, but it can also manifest itself, more subtly, in the various forms of congenital myopathy. The clinical course is slowly progressive and over time patients may develop respiratory insufficiency secondary to the myopathy, and bone and joint deformities such as scoliosis caused by reduced axial muscle strength. Recently a cohort of 21 Italian patients showed a wide spectrum of clinical presentations, including different ages at onset and different patterns of muscle weakness and cardiac involvement [40]. Considering this “blurred clinical-genetic scenario”, the authors suggested that there exist three forms of this type of myopathy: 1) an early-onset distal myopathy with cores, 2) a late-onset form of distal myopathy without histological evidence of cores and with variable association of cardiomyopathy and/or fiber-type disproportion, and 3) the least frequent form consisting of limb-girdle involvement with myofibrillar damage. They also underlined that there is no correlation of mutation sites with severity and that the phenotype cannot be predicted on the basis of the genotype [40, 41].

## Diagnostic approaches

Congenital myopathy is usually suspected on the basis of the clinical presentation, even though, as we have said, this can be highly variable. Although, for this reason, it is extremely difficult to make an accurate diagnosis, this should nevertheless be attempted before running the specific genetic tests that, it is hoped, will establish the correct molecular diagnosis — a crucial objective from the perspective of prognosis, prevention and treatment.

A correct diagnostic approach requires the integration of data from: clinical evaluations (including a detailed family history), muscle biopsy (including histological, immunohistochemical and electron microscopy examinations) and muscle MRI. Serum CK measurement and EMG, which are very useful diagnostic tools in other neuromuscular disorders, are not particularly helpful for diagnosing congenital myopathy, given that CK levels are usually normal or mildly elevated, while EMG can either be normal or, at most, show myopathy-like findings or neuropathic changes, as seen in some cases of nemaline myopathy. Ultrasound images, on the other hand, may be more suggestive, for example in some cases of central core myopathy in which it is possible to appreciate increases in fat or connective tissue; however, even these findings have to be considered largely nonspecific.

Given the complexity of the clinical evaluation of these forms, a diagnostic algorithm is needed that clearly sets out the importance and significance of the various instrumental tests that should be used in order to reach the most accurate diagnosis possible. Today, thanks to the possibility of combining genetic data (including the dataset obtained by massive analysis of multiple genes with methods of next-generation sequencing, NGS) with clinical and morphological findings and MRI results, our chances of achieving a precise diagnosis are increasing all the time [42, 43]. Thus, we here summarize the three major steps, namely histological, myoimaging and genetic, to follow in order to achieve a complete diagnosis in the different groups of congenital myopathies.

## Histological findings in muscle biopsies

**Fig. 1:**
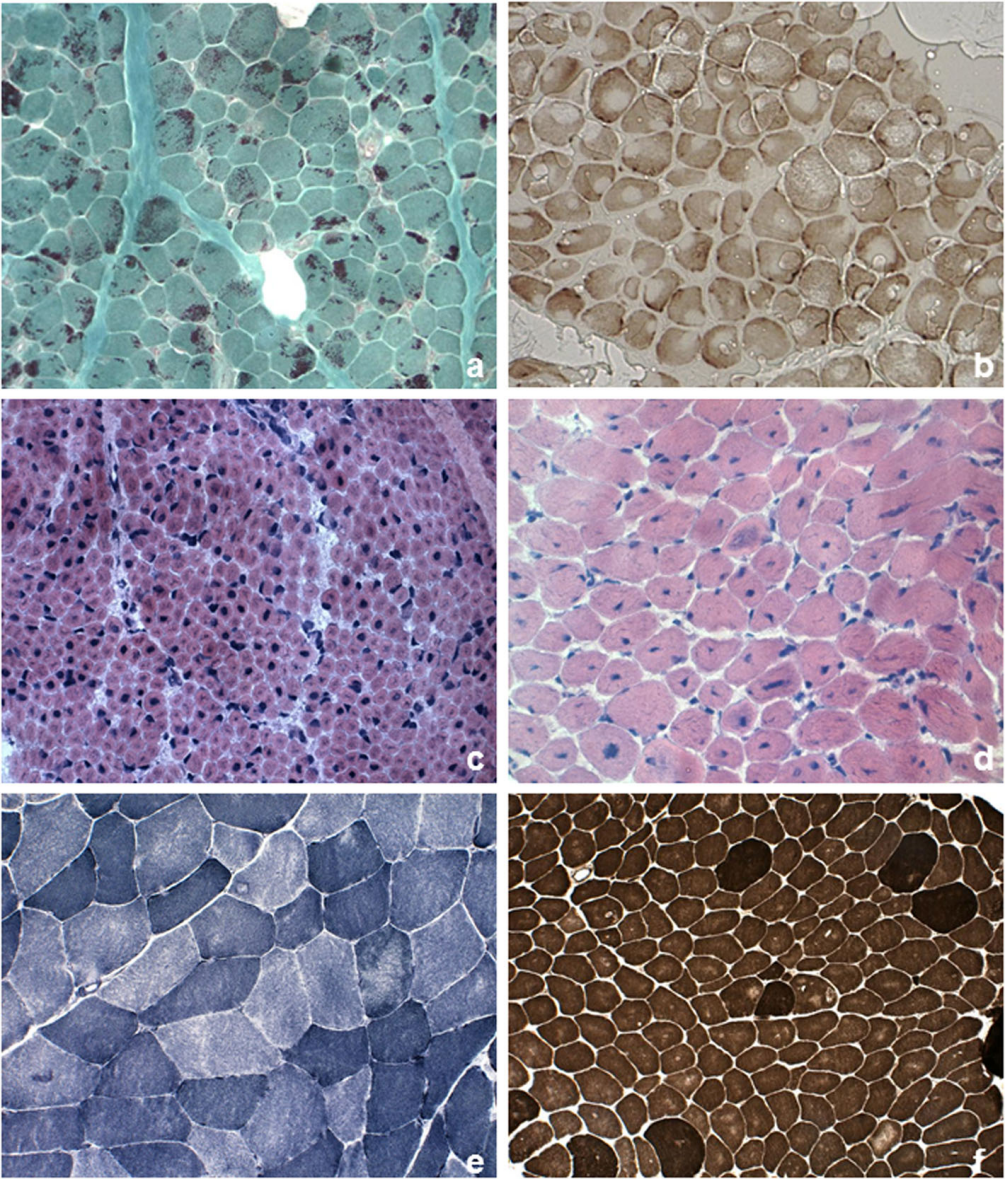
Pathological features in skeletal muscle biopsy observed in congenital myopathies. **a** Nemaline rods. Gomori trichrome stain showing clusters of purple staining rods at the periphery of most fibers and some internal within fibers. **b** Cores. Cytochrome c oxidase stain demonstrating numerous cores of varying size, centrally or peripherally. **c** Central nuclei. Quadriceps biopsy from a boy presenting X-linked myotubular myopathy. Hematoxylin–eosin stain (HE) showed large central nuclei in several fibers. **d** Central nuclei. Quadriceps biopsy from a 5-year-old patient with centronuclear myopathy due to a mutation in *DNM2*. HE demonstrated centrally placed nuclei in the majority of fibers, variation in fiber size and increased connective tissue. **e** Multi-minicores. NADH-TR stain showing areas in both fiber types of varying size and number devoid of oxidative enzyme stain. **f** Congenital fiber type disproportion. ATPase pre-incubated at pH 9,6 stain showing the small size of the light-staining type 1 fibers and type 1 fiber predominance

Figure 1 summarizes major histological findings in congenital myopathy. The histopathological features of the main pathological categories are described below.

### Nemaline bodies

The nemaline bodies (small rod-like inclusions in muscle fibers) that characterize nemaline myopathy are typically visualized by Gomori trichrome staining. Nemaline bodies consist mainly of alpha-actinin, and on electron microscopy they are visible as rod-like or oval-shaped electron-dense structures. They are usually located in the cytoplasm and often cluster at the periphery of muscle fibers; however, they can sometimes be present in the nucleus, and in such cases they are very difficult to identify. Nemaline bodies can also be difficult to identify in extremely young patients, under 12 months of age, in whom the fibers are minute. The number of fibers affected is variable, as is the number of bodies a fiber contains, but these aspects are unrelated to the severity of the phenotype. A wide variation in fiber size may be observed, although type 1 fiber atrophy and type 1 fiber uniformity or predominance are common.

Nemaline bodies may occur in other severe forms of muscle degeneration, both primary and secondary [44], and in healthy muscle during the physiological aging process.

#### Cap myopathy

In this particular form, the muscle fibers contain well demarcated subsarcolemmal cap-like (triangular) inclusions, which appear reddish or purple with the modified Gomori trichrome stain and eosinophilic when stained with hematoxylin and eosin; they show no ATPase activity [44]. Ultrastructural studies show that these caps are present haphazardly on the myofilaments, that the arrangement of the myofibrils in the affected zone does not respect the orientation of the fibers, and that the normal structure of the sarcomere is lacking. The Z-bands are thickened. The structure of the adjacent sarcomeres is normal. Between 4% and 100% of fibers are affected and the proportion affected seems to show a correlation with the severity of the disease and the patient’s age.

#### Zebra body myopathy

Zebra bodies are elongated filamentous structures with thin, dark bans alternating with wider dark ones. Zebra bodies may be a prominent feature of a biopsy and this led to the designation of “zebra body myopathy” but they can be also found in myotendinous junctions and normal extraocular muscle [44].

### Core and minicores

The cores can be single or multiple and they are seen mainly in type 1 fibers. As the disease evolves, there may be more than one core per fiber, even though there are rarely more than two or three. Under electron microscopy, core areas are characterized by the absence of both mitochondria and glycogen, and by a variable degree of destruction of the contractile apparatus; the cores appear structured or unstructured, depending, respectively, on whether or not sarcomeric organization is preserved. Histochemical staining for ATPase activity does not detect structured cores (which are the more frequent), while unstructured ones show up as lighter areas compared with normal fiber areas. Biopsy can show mild variation in fiber diameter, but there are also reports of muscle biopsies revealing not only cores, but also a marked dystrophic pattern and fibrotic tissue replacing muscle tissue [45]. Instead, in very early-onset cases, the formation of the cores may not yet be visible, which seems to suggest an age-dependent pathogenic mechanism. This has been observed in a mouse model of the disease [46].

Like cores, minicores can be detected using stains for oxidative enzymes (NADH, COX and SDH); on cross sections they often appear only as indistinct irregularities in the texture of the muscle tissue or as punctiform areas with no staining. Multiminicore myopathy is characterized by hypotrophic fibers and type 1 fiber predominance. Under electron microscopy, minicores present as focal areas of myofibrillar disruption with absence of mitochondria. Immunohistochemistry may reveal accumulations of proteins such as desmin, myotilin and filamin C, both in central cores and in minicores, although these findings are not indicative of a specific genetic form [47]. Finally, it should be remembered that cores and minicores may be a non-specific finding, given that they can be observed in other conditions, such as denervation, certain metabolic conditions, and even after eccentric exercise in healthy subjects.

### Centronuclear myopathy

The main histopathological feature of X-linked myotubular myopathy is the presence of large central nuclei. These, which can occur physiologically in muscle fibers during the early stages of maturational development, i.e. in embryonal myotubes (with the difference that embryonal myotubes, unlike, mature muscle fibers have yet to differentiate into type 1 and type 2 fibers), have a vesicular appearance and sometimes occupy the whole of the fiber cytoplasm. A striking feature associated with central nuclei is the presence of dark-staining areas under oxidative enzyme stains and PAS staining, reflecting aggregation of mitochondria and glycogen. On staining for ATPases and oxidative enzymes the nuclei appear as optically empty vacuoles. Type 1 fibers may be predominant, and most fibers, particularly the type 1 kind, are hypotrophic [44]. Symptomatic female carriers of myotubular myopathy and benign, late-onset cases have been reported to show characteristic histopathological changes consisting of a reorganization of the myofibers and myofibrillar pattern that results in the presence, detectable under NADH and PAS staining, of dark cords lying just below the fiber perimeter; these fibers have been called “necklace fibers” [27].

In autosomal centronuclear myopathy, the nuclei are not large, but they are often surrounded by a clear halo, where oxidative enzymatic activity is reduced; there can be multiple internal nuclei in myofibers, a finding that allows differential diagnosis versus other forms of myopathy without overt internal nuclei. All the alterations described preferentially affect type 1 fibers which appear smaller, but more numerous (type 1 fiber predominance). Type 2 fibers are usually hypertrophic. The only other typical feature is the presence of “radial strands”, which are probably the result of a disruption of the myofibrillar network. These changes are clearly visible under NADH staining. It is interesting to observe that there is a weak association between genotype and histology, and we recently [25] underlined that radial sarcoplasmic strands, previously described only in late-onset cases, are actually a constant finding in patients with centronuclear myopathy, even when onset occurs in the neonatal period [25].

### Congenital fiber-type disproportion (CFTD)

The original criteria for CFTD [44] stipulated that the type 1 fibers should have a diameter at least 12% smaller than the mean diameter of the type 2A and/or type 2B fibers and that key pathological features of other forms of congenital myopathy (e.g. rods, cores or central nuclei) should be absent. Fiber disproportion can indeed occur in various other conditions, too, including nemaline myopathy and centronuclear myopathy, and the status of CFTD as a distinct nosological entity is therefore still debated. Recent studies suggest that the diameter of type 1 muscle fibers in individuals affected by CFTD is between 40% and 80% smaller than that of type 2 fibers. For this reason, a discrepancy of 35–40% has been suggested as the best criterion for diagnosing CFTD [33]. CFTD is often associated with type 1 fiber predominance. Pathological findings on biopsy may change over time, and in some individuals a diagnosis of CFTD requires a second biopsy.

## Magnetic resonance imaging

In recent years, the application of muscle MRI techniques, also in patients with congenital myopathy, has made a useful contribution to the differential diagnosis of myopathic pictures with overlapping clinical-pathological features [48]. Indeed, muscle MRI can be used to identify, non-invasively, diffuse or localized abnormalities in individual muscle bellies, and thus to identify any known disease patterns and their possible correlations with genetically defined forms [49, 50]; the method can also be used to study patterns of muscle involvement linked to new genetic forms [51, 52]. The high cost of muscle MRI may thus be justified by the fact that its capacity to identify specific disease patterns can open the way for more targeted biochemical and genetic investigations, thereby reducing the use of more expensive laboratory tests. Furthermore, unlike muscle biopsy, this examination, being non-invasive, can be repeated many times without causing patients excessive discomfort.

The first MRI studies in sufficiently large congenital myopathy case series date back to Jungbluth and colleagues’ description of patterns of muscle involvement in central core and nemaline myopathy patients [48]. It has since been shown that the pattern of muscle involvement on MRI can be correlated more specifically with the gene defect than with histopathological changes, and the study of larger populations with specific genetic alterations (see details on genetic determinants below) has made it possible to document more or less selective and diagnostic patterns. In this regard, the pattern of abnormalities found on muscle MRI in patients with mutations in *RYR1* is the most characteristic: the gluteus maximus is the most affected pelvic muscle, whereas in the thigh, there is far greater involvement of the adductor magnus than of the adductor longus, of the vastus lateralis than of the rectus femoris, and of the sartorius than of the gracilis. In mild cases, the first muscle involvement is thought to be that of the sartorius and, to a lesser degree, the adductor magnus. More generally, the muscles of the posterior compartment of the thigh are less affected than those of the anterior compartment. Although muscular degeneration is, overall, less marked in the lower leg than in the thigh, the soleus muscle is usually more affected than the gastrocnemius lateralis, which in turn is more affected than the gastrocnemius medialis. In the anterior compartment, the peroneal group is more affected than the tibialis anterior group [53]. Differently from the above description, patients with mutations in *SEPN1* show, at thigh level, selective involvement of the sartorius, adductor and biceps femoris muscles with relative sparing of the gracilis and rectus femoris; the vastus lateralis, medialis and intermedius, and the semimembranosus and semitendinosus muscles are less affected than other muscles. The muscle most affected in the lower leg is the gastrocnemius, while in the anterior compartment there is appreciable involvement of the extensor muscles of the toes and the peroneal muscles; the impairment of the tibialis anterior and soleus muscles is less marked [49].

Even though, from a clinical perspective, nemaline myopathy is the congenital myopathy most frequently encountered in the general population there have been few studies of genes linked to its different forms. Nevertheless, myopathies associated with mutations in *NEB* have been reported to show selective involvement of the anterior leg compartment muscles, in particular the tibialis anterior, albeit with variable signal intensity, reflecting the clinical severity. Among these cases, the severest clinical phenotype also includes involvement of the rectus femoris, vastus lateralis and gracilis muscles. Myopathies associated with mutations in *ACTA1* are instead characterized by widespread involvement of the muscles of both the thigh and the lower leg, with the exception of the finding of greater involvement of the sartorius compared with the gracilis, and of the soleus compared with the gastrocnemius muscles; once again, the anterior leg compartment muscles (tibialis anterior, peroneal group) are more affected, but in this case involvement of the tibialis posterior can also be noted [54]. The anterior leg compartment muscles are also primarily involved in myopathies related to mutations in *MYH7*, in which the tibialis anterior is the first muscle involved and also the one most affected. This is followed by involvement of the peroneal, soleus and tibialis posterior muscles. The gastrocnemius lateralis is generally spared. The thigh muscles can also be spared, but when they are involved it is the vastus muscles that are affected, whereas the rectus femoris, adductor longus, sartorius and gracilis are always spared. Finally, in patients with mutations in *TMP2* there is evident and widespread involvement of the lower leg muscles and those of the posterior thigh compartment.

In patients with myopathy related to mutations in *DNM2*, muscle MRI often shows predominant involvement of the distal lower leg muscles with early involvement of the plantar flexors [55]. In the thigh there is selective involvement of the adductor longus, semimembranosus, biceps femoris, rectus femoris and vastus intermedius, while the vastus lateralis, vastus medialis, sartorius, gracilis and part of the semintendinosus are spared; in the lower leg there is predominant involvement of the soleus, gastrocnemius medialis and tibialis anterior.

There are fewer myoimaging data available in centronuclear myopathy, probably because these forms are less common and show greater phenotypic severity. That said, it has been shown that the thigh muscles can be spared in forms associated with mutations in *BIN1*, which instead present with predominant involvement of the lower leg muscles, both the gastrocnemius medialis and the anterior compartment muscles, particularly the tibialis anterior (as in cases with *DNM2* mutations), but also the peroneal muscles, whereas (unlike what has been described in *DNM2*-mutated patients) the soleus is preserved. Conversely, the few reported cases with mutations in *MTM1* show severe involvement of the anterior thigh compartment muscles and of the adductor magnus, while the adductor longus, sartorius and gracilis are spared. The lower leg muscle primarily involved is the soleus, while the gastrocnemius medialis is often spared.

Table 1 summarizes major patterns observed in congenital myopathies. To date, most of the studies conducted have selectively investigated the involvement of the lower limbs, although some new research avenues have been explored in myoimaging of congenital myopathies, e.g. the use of a whole-body MR protocol [56, 57]. Moreover, even though the degree of muscle involvement has, for years, been assessed using semi-quantitative criteria [49], in recent times there have been some quantitative analyses, an approach particularly useful for monitoring the degree of disease progression over time and correlating it with the clinical evolution; for the moment, however, longitudinal studies remain scarce [58].

**Table 1:** Muscle imaging findings in congenital myopathies

Muscle Imaging findings	*Gluteus maximus*	*Vastus lateralis*	*Vastus intermedius*	*Vastus medialis*	*Rectus femoris*	*Sartrorius*	*Gracilis*	*Adductor longus*	*Adductor magnus*	*Semimembranosus*	*Semitendinosus*	*Biceps femoris*	*Tibialis anterior group*	*Extensor muscles of the toes*	*Peroneal group*	*Soleus*	*Gastrocnemius lateralis*	*Gastrocnemius medialis*	*Tibilalis posterior group*
*RYR1*	++	+	+	+	-	++	-	+	++		+	-	-		++	++	+	-	
*SEPN1*		+	+	+	-	++	-	++	++	+	+	++	+	++	++	+	++	++	
*NEB*		++	+		++		++		+				++		-	++	-	+	
*ACTA1*		+	+	+	+	++	-	+	+	+	+	+	++	++	++	++	-	-	+
*MYH7*		+	+	+	-	-	-	-					++		+	+	-		+
*TPM2*					-	-	-			+	+	+	+	+	+	+	+	+	+
*DNM2*		-	++	-	++	-	-	++		++	+/-	++	++		-	++		++	-
*BIN1*		-	-	-	-	-	-	-	-	-	-	-	++	+	++	-	-	++	-
*MTM1*		++	++	++	++	-	-	-	++							++		-	

++ = severely affected muscles; + = affected muscles; - = spared muscles

## Genetic determinants and relative genotypes

### Genotypes in nemaline myopathy

The genes classically associated with nemaline myopathy encode sarcomeric thin filament proteins. Mutations in the *NEB* gene encoding nebulin [59], inherited in an autosomal recessive manner, are the most frequent (accounting for over 50%), followed by mutations in *ACTA1* (around 20%) encoding alpha-actin 1 [60], which show an autosomal dominant (90%) or recessive (10%) pattern of inheritance. Some rare cases have been associated with mutations in the alpha-tropomyosin-3 gene (*TPM3*) [61], the beta-tropomyosin-2 gene (*TPM2*) [62], and the troponin T1 gene (*TNNT1*) in the Amish population [63], and more recently also in the gene encoding cofilin-2 (*CFL2*), identified in two related Arab families [64]. This list is expected to grow given that, in recent years, the application of NGS technologies has led to the identification of four new genes potentially involved in this form, namely *KBTBD13, KLHL40, KLHL41* and *LMOD3*. In particular, mutations in *KBTBD13* have been associated with a slowly progressive, childhood-onset form in which axial hypotonia and exercise intolerance are the predominant clinical features. Prolonged muscle contractions have been described in some cases. In this form, it is possible to observe cores as well as the classic nemaline bodies on muscle biopsy [65]. The form associated with mutations in *KLHL40* is relatively frequent and very severe, being characterized by fetal hypokinesia and marked hypotonia. Joint contractures, chest deformities, fractures and respiratory failure can be present at birth. Most patients die in early childhood. Muscle biopsy reveals numerous nemaline bodies, present in practically all large muscle fibers [66].

Mutations in *KLHL41* have been found in five children with widely varying clinical manifestations, ranging from severe forms of fetal akinesia and arthrogryposis, to milder forms with conservation of gait up to 12 years [67]. Finally, in 21 patients from 14 families, Yuen and colleagues recently identified homozygous or compound heterozygous variants in the *LMOD3* gene which encodes leiomodin-3, a protein essential for the organization of thin filaments. Clinically this form is extremely severe: in the majority of patients, alterations are already present in utero; these include polyhydramnios and absence of fetal movements. The condition commonly results in premature birth. Patients do not survive longer than 10 years [68] and most die during the neonatal period due to dysfunction of muscles with bulbar innervation.

### Genotypes in core myopathy

Central core myopathy is classically associated with mutations, dominant or recessive [69, 70], in *RYR1* located on chromosome 19q13.1, which encodes the muscle ryanodine receptor (RYR1), a large protein with a molecular mass of 563.5 kDa that assembles as an oligotetramer and is located in the sarcoplasmic reticulum, in contact with the dihydropyridine receptor (DHPR) and other sarcoplasmic reticulum proteins. RYR1 has a key role in calcium release and electromechanical coupling in muscle [71]. RYR1 shows a complex multimeric organization: the C-terminus serves as the transmembrane domain, but also contains other functional domains such as the binding site of calmodulin; the hydrophilic N-terminal region protrudes into the cytoplasm and constitutes the foot of the calcium channel; the central portion contains several loops in connection with the DHPR and other domains involved in the formation of the channel [72].

Mutations in *RYR1* have been associated with centronuclear myopathies [73] and CFTD [74], and also linked to a susceptibility to malignant hyperthermia (MH) [75], a rare but major complication of anesthesia that manifests itself through a lethal rise in body temperature and tetanic muscle contractions. The mutations that cause congenital myopathy remain, in general, distinct from those responsible for MH susceptibility, which are typically located in the N-terminal region of the gene; different pathogenic mechanisms have been proposed to explain these very diverse clinical manifestations [76]. Nevertheless, it is thought that patients with congenital myopathy due to *RYR1* mutation may also be at risk of MH.

From a genetic perspective, multiminicore myopathy is more heterogeneous. Indeed, it can be caused both by mutations in *RYR1* (in its autosomal recessive form) and by mutations in *SEPN1* [77]. This latter gene encodes selenoprotein N1, another sarcoplasmic reticulum glycoprotein with a molecular weight of di 70 kDa. The selenoproteins perform an important antioxidant function and also have a role in calcium homeostasis. Selenoprotein N deficiency has been associated with satellite cell loss resulting in impaired muscle regeneration [78]. Mutations in *SEPN1* are also responsible for a congenital muscular dystrophy characterized by marked spinal rigidity (rigid spine muscular dystrophy, RSMD), and patients with multiminicore myopathy associated with selenoprotein N deficiency can present clinical features that partially overlap with those of RSMD patients, such as early-onset spinal rigidity and respiratory failure [77].

The literature also reports descriptions of dominant mutations in *MYH7*, the gene encoding the beta myosin heavy chain protein whose mutations have been implicated in cardiac (but also skeletal) muscle disorders. Affected individuals present slowly progressive childhood-onset muscle weakness; this may be distal or proximal and can be associated with cardiorespiratory impairment. A family history of sudden death has been reported in one family [38]. It is estimated that in around 50% of core myopathies no genetic basis has yet been identified [79].

### Genotypes in centronuclear myopathy

X-linked myotubular myopathy is associated with mutations in *MTM1*, which encodes myotubularin, a 603-amino acid phosphatidylinositol 3-phosphate (PI3P) phosphatase. Myotubularin is a ubiquitous nuclear protein involved in the transduction of signals between the nucleus and cytoplasm, and probably related to cell differentiation and cell maturation [80].

Autosomal dominant centronuclear myopathy is classically associated with mutations in *DNM2* which codes for dynamin 2 [81], a GTPase that is involved in the mechanisms of endocytosis and transport of organelles along the network of microtubules, and also plays a key role in centrosome formation [82]. Variants of this gene have also been identified in a form of dominant Charcot-Marie Tooth disease associated with cataracts [83], while homozygous mutations have recently been described in a Pakistani family with a lethal form of congenital arthrogryposis [84].

The *BIN1* gene on chromosome 2q14 is, instead, responsible for an autosomal recessive form of centronuclear myopathy [85], and has recently also been associated with an autosomal dominant or sporadic adult form [86]. *BIN1* encodes amphiphysin 2, a protein that has a key role in the mechanisms of exocytosis and interacts with dynamin to bring about correct positioning of the core inside the muscle fiber and the regular formation of T tubules [87]. Recessive mutations in *BIN1* were described in a small group of Indian and Moroccan patients with quite a severe phenotype, generally present at birth (just one childhood-onset case was reported), and with a mortality rate of 40%. Its clinical characteristics included marked muscle atrophy and concomitant cardiomyopathy in some cases; serum CK could be raised. No respiratory involvement was reported. The autosomal dominant or sporadic form due to mutations in *DNM2*, found in the European population, is reported to be far less severe and to have a very late onset, between 20 and 50 years of age. In these cases, too, there is no respiratory involvement, but affected individuals can present ptosis and ophthalmoparesis.

Mutations in *RYR1*, known to be associated with core myopathy, seem to be another common cause of centronuclear myopathy and have been described in cases exhibiting variable phenotypes, which generally include a neonatal onset with joint contractures and axial hypotonia [88]. In addition to centrally located nuclei, biopsies in these patients can also reveal the cores characteristically associated with mutations in *RYR1*. Less commonly, mutations in *TTN*, which encodes titin, a giant sarcomeric protein [89], in *SPEG*, which encodes a Z-band protein that shows a strong interaction with *MTM1* and also results in dilated cardiomyopathy [90], and in *MYF6* and *CCDC7* have been reported in a few patients with clinical features reminiscent of centronuclear myopathy [91].

### Genotypes in congenital fiber-type disproportion (CFTD)

Mutations in the alpha-tropomyosin-3 gene (*TPM3*), which may be inherited in an autosomal dominant or recessive fashion, are the most common cause of CFTD [92, 93]. In almost all cases, they cause a mild form of the disease. Indeed, affected patients retain the ability to walk until adulthood. Recessive mutations in *RYR1* are the second most common cause of CFTD (accounting for approximately 20% of cases). The finding of a marked fiber size disproportion (greater than 30–40%) on muscle biopsy may be suggestive of this form [74]. CFTD can also be caused by other gene mutations showing an autosomal recessive (*SEPN1)* or dominant (*ACTA1*, *TPM2* and *MYH7)* pattern of inheritance [37, 94, 95].

### Genotypes in the most recently described forms

Recently Schartner and colleagues [96] described a novel class of congenital myopathy associated with recessive and dominant mutations in *CACNA1S*, the pore-forming subunit of DHPR located on the T-tubule in the proximity of a Ca2+ release channel (ryanodine receptor; *RYR1*) on the sarcoplasmic reticulum. Patients presented perinatal hypotonia, severe axial and generalized weakness and ophthalmoplegia. Analysis of muscle biopsies demonstrated a characteristic intermyofibrillar network (due to sarcoplasmic reticulum dilatation), internal nuclei, and areas of myofibrillar disorganization.

Mutations in *STAC3* cause an autosomal-recessive disorder found in the Lumbee Native American population, characterized by clinical features including congenital onset of muscle weakness, multiple joint contractures, dysmorphic facial features, and susceptibility to malignant hyperthermia. Patient muscle biopsies revealed a non-specific myopathic pattern. *STAC3* protein (SH3 and cysteine-rich domain 3) is an essential component of the excitation-contraction coupling apparatus [97, 98].

Recessive mutations in *SCN4A*, encoding the α-subunit of the skeletal muscle voltage-gated sodium channel, were recently associated with a novel form of congenital myopathy. Affected subjects presented variable clinical features including fetal hypokinesia or association of the myopathy with congenital hypotonia, significant respiratory and swallowing difficulties, spinal deformities, and facial weakness with ptosis. Muscle biopsies showed myopathic features including fiber size variability and the presence of fibrofatty tissue of varying severity, without specific structural abnormalities [99, 100]. Mutations in *SCN4A* have previously been described in hypokalemic or hyperkalemic periodic paralysis, paramyotonia, myotonia congenita, and myasthenic syndrome. Finally, recessive mutations in *SPTBN4*, encoding βIV-spectrin, a non-erythrocytic member of the β-spectrin family, were reported in a family associated with congenital myopathy, neuropathy, and central deafness [101].

## The use of massive parallel sequencing to tackle heterogeneity in congenital myopathies

Massive next-generation sequencing (NGS) is a technique involving the parallel sequencing of DNA molecules that are spatially separated in a flow cell. It allows the sequencing, in a single run, of between hundreds of millions and billions of base pairs of DNA, depending on the type of NGS technology being used. NGS can be used for different applications, such as genomic analysis, whole exome sequencing (WES) targeting the entire set of human exons (about 1.5% of the human genome), transcriptome sequencing and metagenomic applications. The application of NGS platforms generates an unprecedented amount of information, and this makes the management, storage and, above all, analysis of the data a real challenge [102]. This mass of data is such that an interconnected system (data analysis pipeline) with a very high operational capacity is required to allow its storage, management and processing [103]. The accuracy of NGS platforms derives from the repeated sequencing of a given region of interest using a massive and parallel process in which each sequence contributes to the depth of “coverage”, and makes it possible to obtain a consensus sequence. Excellent reviews, including [103], illustrate the pros and cons of this innovative DNA sequencing approach in clinical settings.

The impact of NGS platforms in the field of basic research has been considerable and suggests that this technology will prove widely applicable. In recent years, NGS has been increasingly used for the genetic diagnosis of diseases that, like the different forms of congenital myopathy, have heterogeneous clinical phenotypes and involve multiple genes. Congenital myopathy is indeed an area in which overlapping skeletal muscle biopsy findings, the lack of specific clinical presentations, and genetic heterogeneity can all complicate the molecular diagnostic process to the point that even expert clinicians often struggle to make an accurate diagnosis. For this reason, the use of massive parallel sequencing is useful in order to reach, rapidly and inexpensively, a definitive diagnosis. Recent testing of panels of disease genes using massive NGS approaches has given different findings. In particular, it has been emphasized that the diagnosis of congenital myopathy is highly dependent on interpretation of the muscle histology [104], whose features can point to a certain type of congenital myopathy, but are not necessarily gene specific. More than 20 loci have been linked to the different forms, including three exceptionally large genes (*TTN, NEB* and *RYR1*), and this, in itself, is a real challenge for molecular diagnosis. Oliveira and colleagues developed a new massive parallel sequencing approach that allowed them to simultaneously analyze 20 congenital myopathy-related genes. Results obtained using this approach led to the identification of pathogenic variants in 70% of the patients in their study — this is a large proportion given the considerable genetic and phenotypic heterogeneity of congenital myopathy — and thus demonstrate the value of this approach. Similar approaches by other groups have given heterogeneous results.

Published data demonstrate that more than 40% of patients affected by an limb-girdle muscular dystrophy or congenital myopathy usually obtain a molecular diagnosis thanks to the use of NGS strategies [105]. The rate of diagnosis in patients with a distal myopathy or with an isolated hyperCKemia is lower. NGS data show that a large proportion of patients (20–30%) have mutations in genes typically associated with other forms of inherited myopathy and not traditionally linked to the observed phenotype [106]. This finding may be taken as further proof of a clear and strong clinical overlap between different skeletal muscle disorders, and it broadens the spectrum of disease presentations associated with many of the congenital myopathy genes. Moreover, using WES approach the rate of diagnosis in patients with neuromuscular disorders ranges from 16% to 47% [105, 107]. Genome sequencing has not been applied in the diagnostic setting so far: its cost and, above all, the challenges related to the interpretation of the molecular findings still preclude its routine diagnostic use [108].

Although the use of NGS strategies represents a modern approach to “solve the unsolved” [109], caution is still required in the interpretation of massive data. Most of the inherited myopathies in childhood are clinically heterogeneous and show overlapping phenotypes; with a dramatic technical progress and a cost-effective strategy at hand, many parents and clinicians now decide for gene tests as first line diagnostics [110]. However the “excess of pathogenic variants” does not facilitate sorting out an ultra-rare genetic noise from disease causative changes and “synergistic heterozygosity” in the pathogenesis of the disorder is far to be demonstrated in neuromuscular disorder. Thus, there is a tremendous need (now more than ever) for truthful definition of clinical phenotypes using new Phenomics approaches [111] as well as new consensus and shared criteria in muscle biopsy interpretations.

## Therapeutic considerations

With the exception of the aggressive, very early-onset forms, congenital myopathy generally has a benign clinical course and prognosis. There currently exist no definitive treatments for all the forms of congenital myopathy, and it is therefore necessary to plan appropriate rehabilitation care as well as correct management of cardiac, respiratory and swallowing problems in order to preserve patients’ basic functions, counteract complications and ensure a good quality of life [112, 113].

In principle, it remains important to establish the genetic basis of the disease. The identification of mutations in certain genes has implications for both prognosis and prevention. For example, respiratory monitoring is particularly important in patients with mutations in *NEB*, *ACTA1* or *SEPN1*, and cardiac disease prevention in patients harboring mutations in *MYH7* or *TPM2* [105].

One feature shared by the different forms of congenital myopathy is that patients tend to show respiratory muscle impairment, which may be due either to weakness of the intercostal muscles or to deformities associated with scoliosis. In some cases progressive respiratory failure occurs even when limb strength is preserved. Nocturnal hypoventilation seems to be particularly common in young adult patients and this condition, if untreated, can cause sudden death. A forced vital capacity corresponding to 60 % of the predicted value is a useful threshold, below which studies should be performed to ascertain whether the patient is affected by nocturnal hypoventilation.

Several case reports and studies on small populations document the effectiveness of a beta2 agonist (albuterol/salbutamol) as a means of boosting exercise resistance in forms of congenital myopathy with early fatigability; as a secondary effect, this drug also favors bronchodilation [114]; unfortunately, however, since the respiratory insufficiency in these forms is due to the inability of the inspiratory muscles to expand the rib cage, salbutamol has little effect in this regard.

Recent research has offered new opportunities for therapies and new information is now accessible to patients and physicians (see URL https://clinicaltrials.gov/ct2/results? cond=Congenital+Myopathy&term = &cntry1 = &state1 = &recrs=). Specifically, there are there are attempts to modulate calcium release from the sarcoplasmic reticulum in central core disease due to mutations in *RYR1* (using dantrolene, 5-aminoimidazole-4-carboximide ribonucleoside, and 1,4-benzothiazepine derivatives JTV519 and S107) and also to correct the abnormalities of oxidative metabolism (with N-acetylcysteine) [112]. In XLMTM, there are new opportunities by applying in a clinical setting AAV8-based gene therapy already known to improve the clinical and histopathological phenotype in *Mtm1*-knock-out mice, and to increase muscle strength and survival in a canine model [112]. Most orthopedic abnormalities in congenital myopathy are quite amenable to conservative or surgical treatment. In patients with severe scoliosis, an early surgical approach may be recommended in order to better preserve respiratory function. Achilles tendon contractures can develop in patients with distal involvement and can sometimes necessitate surgical lengthening of the tendon.

Physical therapy to maintain proper joint function is often useful. Possible options, in the most severe cases, are insoles or braces to stabilize gait and maintain the upright position for as long as possible. It has, in fact, been shown that conserving gait and the upright position can delay the onset of spinal deformity.

Although evidence that patients with congenital myopathy benefit from specific physiotherapy exercise regimens is scarce [115], it is known that regular aerobic exercise, such as cycling, hydrotherapy and, when possible, swimming, is advantageous. These patients may have an increased risk of osteoporosis and bone fractures. Calcium supplementation and the maintenance of adequate levels of vitamin D are therefore recommended. Bone mineral density assessments may identify patients at increased risk of osteopenia. Cardiomyopathy is rarely associated with congenital myopathy, but it is a potential issue in the presence of mutations in certain genes such as *TTN* and *MYH7*. It may therefore be advisable to perform a cardiac examination with echocardiogram or electrocardiogram every 2–3 years in childhood, and every 3–5 in adulthood, or more frequently if clinical manifestations appear. The potential use, in some forms, of antioxidant therapy, such as ubidecarenone, to prevent cardiac or respiratory muscle dysfunction is debated [116].

## Conclusions

In this manuscript, we reviewed clinical and genetic forms of congenital myopathies and defined possible histological and imaging characteristics that can improve cost-effectiveness in diagnosis if carefully balanced in the clinical setting. While detecting congenital myopathy appear to be easy, establishing its correct genetic diagnosis is not. Indeed, there is no direct link between specific, histologically defined forms of congenital myopathy and their genetic cause, as many of the histological patterns can actually be caused by mutations in different genes, while mutations in a single gene can result in various histopathological abnormalities (Table 2). Thus, it is important to classify patients correctly and the suggested use of two broad categories based on age at onset: neonatal and infancy onset and later childhood onset appear to provide a first guide in prioritizing genetic testing in affected patients [1]. To date, applications of NGS methodologies (especially targeted gene panels of multiple genes) seem to be the best approach to adopt in the years to come in the diagnosis of congenital myopathy, as it allows lower costs and shorter times of analysis compared with traditional Sanger sequencing, especially considering the large size of genes such as *NEB, TTN* and *RYR1*. As in many NGS studies, the real challenge, however, will be to succeed in distinguishing true pathogenic mutations from population polymorphisms, and to achieve functional validation of possible new genes/ mutations. It is also necessary to appreciate that single patients may be found to carry different mutations in multiple genes, and that some of these may play a role in the expression of the phenotype (gene modulators). The clinical use of NGS multigene testing, however, should be reserved to a step when clinical data and imaging or histological evidences in muscle have already been collected and carefully classified. Genotype without deep phenotype, indeed, might prove useless and hinder pinpointing the true gene rather than accelerate the “diagnostic odyssey”. Too rapid genotyping may produce several false positive or increase the number of genetic variants whose pathogenic significance needs to be functionally corroborated.

**Table 2:**
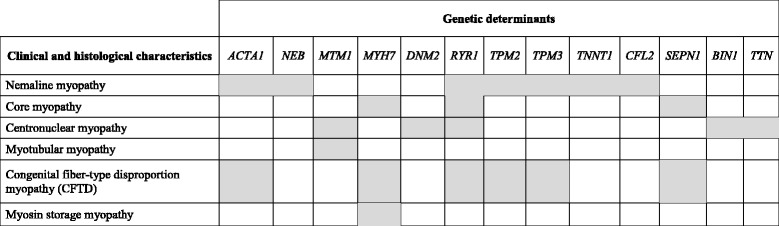
Genetic heterogeneity in congenital myopathies

Grey boxes indicate genes associated with specific clinical and morphological phenotypes. Only “more common” genes are indicated

Identification of the true gene mutation [41] is the prerequisite for more precise clinical stratification. Although curative opportunities are scarce, emerging evidences of new therapeutic strategies, including gene transfer in X-linked myotubular myopathy, enzyme replacement in MTMR14-related congenital myopathies, anti-atrophy approaches, in addition to upregulation of the fetal isoform of skeletal muscle a-actin in patients with recessive mutations in *ACTA1* [66, 117] open new chances and challenges. The significant overlap between genetic and structural subgroups of the congenital myopathies suggest that possible therapies may be operative in a broad range of conditions and perhaps also benefit a range of patients beyond the limits of their clinical syndromes [118]. Overall, this demands for an even more accurate stratification of patients and collection of meaningful outcome measures for future trials.
